# Phylogenetic analysis and genetic diversity of the xylariaceous ascomycete *Biscogniauxia mediterranea* from cork oak forests in different bioclimates

**DOI:** 10.1038/s41598-022-06303-7

**Published:** 2022-02-16

**Authors:** Daniela Costa, Vitor Ramos, Rui M. Tavares, Paula Baptista, Teresa Lino-Neto

**Affiliations:** 1 Centre of Molecular and Environmental Biology (CBMA), Department of Biology, University of Minho, Campus de Gualtar, 4710-057 Braga, Portugal; 2 Centro de Investigação de Montanha (CIMO), Instituto Politécnico de Bragança, Campus de Santa Apolónia, 5300-253 Bragança, Portugal

**Keywords:** Genetic variation, Phylogenetics, Pathogens, Fungal evolution

## Abstract

Cork oak is a tree species with ecological importance that contributes to economic and social development in the Mediterranean region. Cork oak decline is a major concern for forest sustainability and has negative impacts on cork oak growth and production. This event has been increasingly reported in the last decades and seems to be related with climate changes. *Biscogniauxia mediterranea* is an endophytic fungus of healthy cork oak trees that turns into a pathogen in trees weaken by environmental stress. Understanding the drivers of *B.* *mediterranea* populations diversity and differentiation is expected to allow a better control of cork oak decline and preserve forest sustainability. Endophyte isolates from different cork oak forests were identified as *B.* *mediterranea* and their genetic diversity was evaluated using phylogenetic and microsatellite-primed PCR analyses. Genetic diversity and variability of this fungus was correlated with environmental/phytosanitary conditions present in forests/trees from which isolates were collected. High genetic diversity and variability was found in *B.* *mediterranea* populations obtained from different forests, suggesting some degree of isolation by distance. Bioclimate was the most significant effect that explained the genetic variability of *B.* *mediterranea*, rather than precipitation or temperature intensities alone or disease symptoms. These findings bring new implications for the changing climate to cork oak forests sustainability, cork production and quality.

## Introduction

Cork oak (*Quercus suber* L.) is an evergreen oak species with high economic, ecological, and social importance in the Mediterranean region^1,2^. This species is mainly distributed throughout the Mediterranean Basin, where is well adapted to the climate. Despite that, global climate changes can induce abiotic stress on trees, impacting negatively cork oak growth and productivity^3,4^. Indeed, cork oak decline has been increasingly reported in the last decades and seems to be linked to climate changes, which can increase susceptibility to pathogen attack and facilitate infection by opportunistic pathogens^5,6^. *Biscogniauxia mediterranea* is an opportunistic pathogen with an endophytic lifestyle that has been associated with cork oak decline in the Mediterranean region^7–9^. This fungus causes charcoal disease that leads to an extensive inner bark and xylem necrosis, often associated with a blackish exudation on the outer bark^10,11^. As the appearance of *B.* *mediterranea*-related symptoms are mainly associated with weakened trees (*e.g.,* by drought), the incidence of this disease increased over the last years and was recently reported on young trees^12,13^.

Molecular markers are widely used to study population genetics of phytopathogenic fungi^14^. Early studies on *B.* *mediterranea* populations used random amplified polymorphic DNA (RAPD), being able to detect a high genetic variability for geographically close populations^9,15^. These results indicated a high rate of sexual reproduction and heterothallic mating system displayed by this species. More recently, high genetic variability was reported within a single stromata using microsatellite-primed PCR primers^16^. Also, *B.* *mediterranea* populations from different Mediterranean countries displayed high genetic diversity (by using sequence analysis of Internal Transcribed Spacer of ribosomal RNA gene, translation elongation factor 1-α and β-tubulin, as well as microsatellite-primed PCR primers)^17^, but studied isolates were not phylogenetic associated with host species or age, geographic position, or charcoal canker expression. However, the analysis of Tunisian populations of *B.* *mediterranea* by using microsatellite-primed PCR primers suggested a positive association of their morphological variability and ecological factors, such as altitude, rainfall, and temperature^18^. Altogether, these findings pointed to the high plasticity and adaptability of this species to the environment^15,17^. In this work, we aimed to understand the correlation of *B.* *mediterranea* genetic populations with environmental factors and disease symptoms found on cork oak trees from different bioclimates. For increasing the significance of molecular characterization of *B.* *mediterranea* populations we used two different typing analyses, a multilocus (6 loci) and a microsatellite (4 primers) approach. This work will increase the current knowledge about drivers of *B.* *mediterranea* genetic variability and contribute for the formulation of appropriate disease management.

## Results

The molecular characterization of *B. mediterranea* endophytic isolates from cork oak trees was performed using a multilocus sequence analysis and microsatellite-primed PCR fingerprinting. From all analyzed loci, partial glutamine synthetase (*GS*) revealed the highest variability with 19.2% of identical sites (including the outgroup sequences, *B.* *atropunctata*, *B.* *nummularia* and *Xylaria hypoxylon*), followed by actin (*ACT*; 27.6%), translation elongation factor 1-α (*TEF*; 53.2%), β-tubulin 2 (*TUB2*; 57.7%), chitin synthase 1 (*CHS*; 61.4%) and internal transcribed spacer (*ITS*; 66%). As expected, the nucleotide datasets displayed higher percentage of identical sites without outgroups. The corresponding values ranged from 36.9% (*GS*) to 94.7% (*TUB2*). The multilocus alignment was in line with this trend (23.4% of identical sites with outgroups; 66.4% without outgroups).

**Figure 1:**
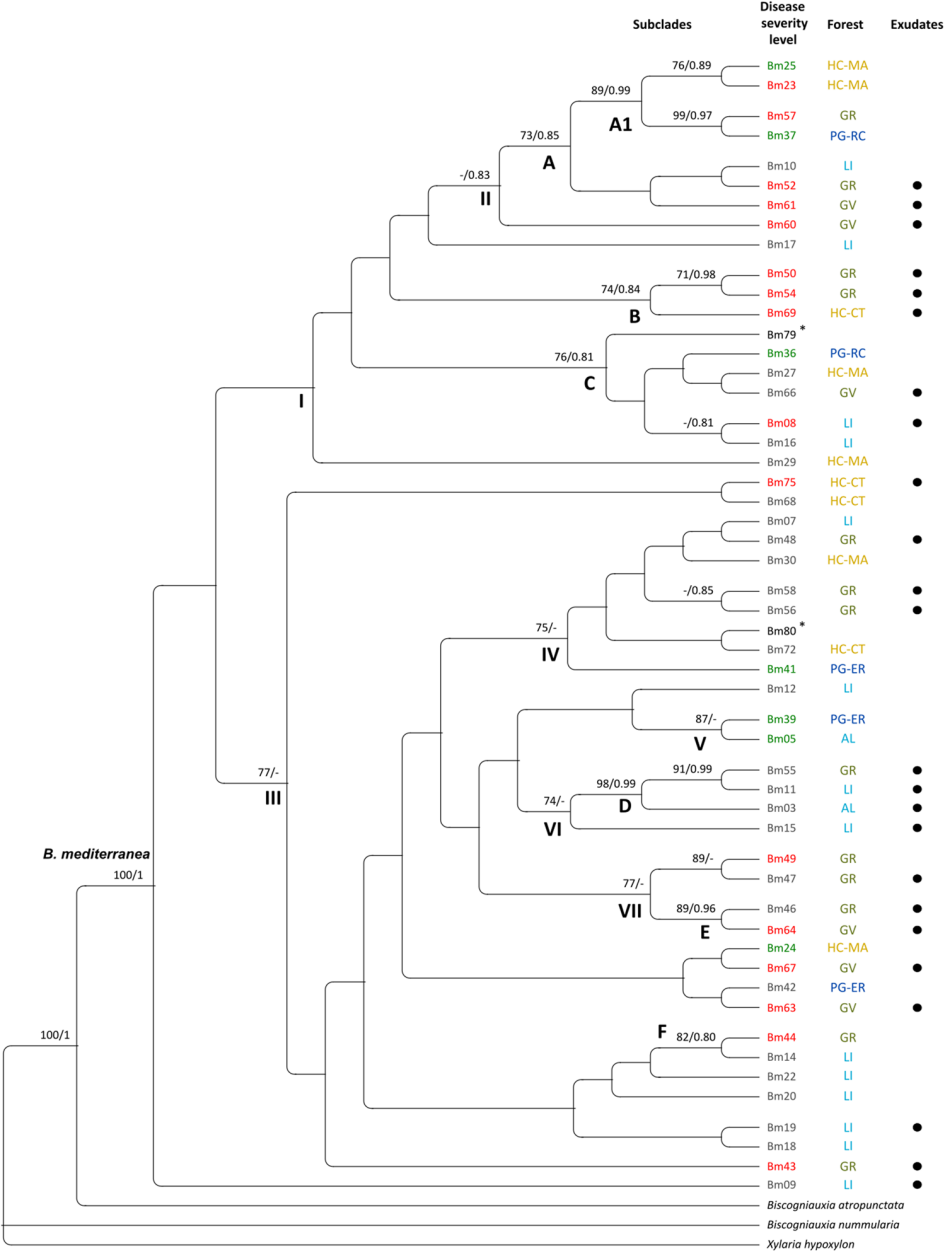
Cladogram showing the phylogenetic relationship between the 52 *B. mediterranea* isolates (50 from cork oaks and 2 from olive trees*) and three outgroup species (*B.* *atropunctata*, *B.* *nummularia* and *Xylaria hypoxylon*). This multilocus ML tree was constructed from the concatenated alignment of *ITS*, *TEF*, *GS*, *ACT*, *CHS* and *TUB2* sequences. Numbers shown at nodes are support branch values for ML bootstraps (only BS values ≥ 70 are shown) and BI posterior probabilities (only PP values ≥ 0.8 are shown), respectively. Disease severity levels are highlighted in green, grey, or red, representing isolates collected from healthy, mild, and declining trees, respectively. Black dots refer to isolates collected from trees showing exudation. Forests are highlighted in different colors, according to their bioclimate, as follow: dark blue for hyper-humid (PG-RC and PG-ER), light blue for humid (AL and LI), green for sub-humid (GV and GR) and dark yellow for semi-arid (HC-MA and HC-CT). Clades (A–F) and some groups of sequences (I–VII) are exposed for clarity.

Phylogenetic trees of each single locus were not able to individually resolve *B.* *mediterranea* isolates for any factors of interest (forest, bioclimate, disease severity index or disease symptoms; results not shown). The concatenated dataset comprised sequences from 52 *B.* *mediterranea* isolates and from the three outgroup species (*B.* *atropunctata*, *B.* *nummularia* and *Xylaria hypoxylon*). From a total of 2874 characters, 1775 were constant, 815 parsimony-uninformative and 284 parsimony-informative. Maximum Likelihood (ML) tree produced clades of *B.* *mediterranea* isolates with moderate [ML bootstrap (BS) 70–80; BI posterior probability (PP) 0.8–0.9], high (BS 81–95; PP 0.91–0.95) or very high (BS > 95; PP > 0.95) support values^19,20^, but no evident patterns regarding factors of interest were exposed using this approach (Fig. 1). Isolates obtained from cork oaks growing in different locations and distinct bioclimates did not clustered together; and visible tree disease symptoms did not contribute for the distribution of isolates. For example, Clade A comprised isolates (7; *e.g.* Bm25, Bm57, Bm37) from different forests and bioclimates (from the most humid to the driest), also presenting isolates obtained from trees with different disease severity levels. The same was observed for Clade C (with 6 isolates; *e.g.* Bm36, Bm66, Bm08), which even included an isolate (Bm79) obtained from an olive tree. Moreover, some information can be inferred from the tree. Group III (with moderate ML support) displayed a higher number of isolates from trees with mild symptoms (21 out of the 31 found in Group III, contrasting with 7 isolates in the remaining 19). Clade B (in Group I) only included isolates (Bm50, Bm54 and Bm69) from declining trees, while Clade D (Group III) only included isolates (Bm55, Bm11 and Bm03) collected from mild diseased trees, though both clades presented isolates from different forests and bioclimates. Overall, isolates from different locations were distributed along the phylogenetic tree, with few of them being placed together. The same distribution pattern was observed regarding isolates from different bioclimates, even though the sequences in Group VII refer to isolates (Bm49, Bm47, Bm46 and Bm64) obtained from two forests (GV and GR) with the same bioclimate (sub-humid). Also, a clear distribution pattern was not observed concerning the presence of exudates on the *B.* *mediterranea* host trees. However, subclade A1 only encompassed isolates (Bm25, Bm23, Bm57 and Bm37) obtained from cork oak trees not producing trunk exudates. The opposite was observed for Clade B (Bm50, Bm54 and Bm69) and Group VI (Bm55, Bm11, Bm03 and Bm15), where all isolates were recovered from trees producing exudates, even if from dissimilar bioclimatic locations (sub-humid and humid). In line with the low resolution of *B.* *mediterranea* isolates by any of the studied factors (forest, bioclimate, disease severity index or disease symptoms), the majority of *B.* *mediterranea* isolates collected from the same tree were not clustered together. For instance, although pairwise identity within those isolates was high, isolates from a single tree, such as Bm60, Bm61, Bm63 and Bm67 (pairwise identity of 95.4%) and Bm46, Bm56 and Bm58 (99.6%), were found to cluster better with isolates obtained from other forests and/or were placed apart in the phylogenetic tree. Similarly, isolates collected on olive trees (Bm79 and Bm80) were distantly placed from each other, in separate lineages.

Microsatellite-primed PCR fingerprinting of 68 *B.* *mediterranea* isolates generated different banding patterns. Primer *(GTG)*_*5*_ generated 15 bands (from 0.25 to 1.5 kb)*, (CAG)*_*5*_ generated 17 bands (ranging from 0.3 to 2 kb, in which one was monomorphic), *(ACAC)*_*5*_ generated 17 bands (from 0.25 to 1 kb) and *M13* generated 26 bands (from 0.15 to 1.5 kb). The monomorphic band was removed and a binary dataset with the remaining (74) band positions was used for molecular analysis. Using this approach, the genetic diversity of *B.* *mediterranea* varied within different factors of interest/populations (Table 1). Considering total populations, the number of alleles (*Na*) varied from 1.40 to 1.97 and the number of effective alleles (*Ne*) from 1.45 to 1.57, which were found considering ‘forest’ and ‘exudates’ factors, respectively. These results were corroborated by the genetic diversity found within populations (*Hs*; Table 2). ‘Exudates’ factor (total population) also displayed the highest Shannon’s information index (*I* = 0.50), Nei’s gene diversity (*h* = 0.33) and percentage of polymorphic loci (PPL = 98.7), while ‘forest’ factor shown the lowest genetic diversity (*I* = 0.38; *h* = 0.26; PPL = 67.9). In addition to ‘exudates’, other disease-related factors also revealed high genetic diversity levels for total populations, in particular when considering ‘cankers’ and ‘disease severity levels’ factors (Tables 1 and 2). The genetic diversity of *B.* *mediterranea* also differed among populations when considering individually each environmental/disease-related factor (Table 1). Considering the ‘bioclimate’ factor, isolates obtained from sub-humid forests revealed the highest genetic diversity and those from hyper-humid the lowest. While Grândola (GR, a sub-humid forest) was the forest with the highest *B.* *mediterranea* genetic diversity, PG-ER (hyper-humid) and AL (humid) were the forests with the lowest diversity. Isolates collected from healthy trees (low disease severity level) revealed the lowest genetic diversity and declining trees were associated with more diverse *B.* *mediterranea* isolates. This result is in line with the highest genetic diversity found among *B.* *mediterranea* isolates collected from trees producing trunk exudates. However, the opposite was found for trees with trunk cankers, in which higher genetic diversity was associated with isolates from trees without this symptom. Regarding defoliation, the isolates obtained from trees with very accentuated damage were also less genetically diverse, while those from trees with moderate and light damages presented higher genetic diversity.

**Table 1:** Genetic diversity of *B. mediterranea* populations.

Population	*Na*	*Ne*	*I*	*h*	PPL
**Bioclimate**					
Hyper-humid	1.64 ± 0.09	1.51 ± 0.04	0.44 ± 0.03	0.30 ± 0.02	81.1
Humid	1.76 ± 0.07	1.47 ± 0.04	0.43 ± 0.03	0.28 ± 0.02	86.5
Sub-humid	1.97 ± 0.03	1.59 ± 0.04	0.51 ± 0.02	0.34 ± 0.02	98.7
Semi-arid	1.73 ± 0.08	1.52 ± 0.04	0.47 ± 0.03	0.31 ± 0.02	86.5
Total	1.77 ± 0.04	1.52 ± 0.02	0.46 ± 0.01	0.31 ± 0.01	88.2
**Forest**					
PG-ER	0.91 ± 0.11	1.32 ± 0.05	0.26 ± 0.04	0.18 ± 0.03	40.5
PG-RC	1.47 ± 0.10	1.50 ± 0.04	0.42 ± 0.03	0.28 ± 0.02	73.0
LI	1.62 ± 0.09	1.46 ± 0.04	0.41 ± 0.03	0.27 ± 0.02	79.7
AL	0.99 ± 0.11	1.33 ± 0.05	0.27 ± 0.04	0.18 ± 0.03	43.2
GV	1.51 ± 0.09	1.48 ± 0.04	0.41 ± 0.03	0.28 ± 0.02	74.3
GR	1.92 ± 0.05	1.56 ± 0.03	0.50 ± 0.02	0.33 ± 0.02	96.0
HC-CT	1.42 ± 0.10	1.47 ± 0.04	0.40 ± 0.03	0.28 ± 0.02	68.9
HC-MA	1.39 ± 0.10	1.45 ± 0.04	0.38 ± 0.03	0.26 ± 0.02	67.6
Total	1.40 ± 0.04	1.45 ± 0.02	0.38 ± 0.01	0.26 ± 0.01	67.9
**Disease severity level**					
Healthy	1.70 ± 0.08	1.54 ± 0.04	0.46 ± 0.03	0.31 ± 0.02	85.1
Mild	2.00 ± 0.00	1.55 ± 0.04	0.50 ± 0.02	0.33 ± 0.02	100
Declining	1.97 ± 0.03	1.59 ± 0.04	0.52 ± 0.02	0.34 ± 0.02	98.7
Total	1.89 ± 0.03	1.56 ± 0.02	0.49 ± 0.01	0.33 ± 0.01	94.6
**Defoliation**					
Very accentuated damage	0.97 ± 0.11	1.41 ± 0.06	0.28 ± 0.04	0.20 ± 0.03	40.5
Accentuated damage	1.60 ± 0.09	1.53 ± 0.04	0.45 ± 0.03	0.30 ± 0.02	77.0
Moderate damage	1.92 ± 0.05	1.56 ± 0.04	0.50 ± 0.02	0.33 ± 0.02	96.0
Light damage	2.00 ± 0.00	1.56 ± 0.04	0.50 ± 0.02	0.33 ± 0.02	100
No damage	1.57 ± 0.10	1.53 ± 0.04	0.45 ± 0.03	0.30 ± 0.02	78.4
Total	1.61 ± 0.04	1.52 ± 0.02	0.43 ± 0.01	0.29 ± 0.01	78.4
**Exudates**					
Yes	2.00 ± 0.00	1.58 ± 0.04	0.51 ± 0.02	0.34 ± 0.02	100
No	1.95 ± 0.04	1.55 ± 0.04	0.49 ± 0.02	0.33 ± 0.02	97.3
Total	1.97 ± 0.02	1.57 ± 0.03	0.50 ± 0.02	0.33 ± 0.01	98.7
**Cankers**					
Yes	1.76 ± 0.08	1.55 ± 0.04	0.47 ± 0.03	0.32 ± 0.02	87.8
No	2.00 ± 0.00	1.57 ± 0.04	0.51 ± 0.02	0.33 ± 0.02	100
Total	1.88 ± 0.04	1.56 ± 0.03	0.49 ± 0.02	0.33 ± 0.01	93.9

*Na* represents the number of alleles, *Ne* the effective number of alleles, *I* the Shannon’s information index, *h* the Nei’s gene diversity and PPL the percentage polymorphism loci.

**Table 2:** Genetic differentiation coefficients of *B.* *mediterranea* populations.

Population	*Ht*	*Hs*	*Gst*	*Nm*
Bioclimate	0.341 ± 0.018	0.308 ± 0.015	0.097	4.670
Forest	0.339 ± 0.019	0.258 ± 0.011	0.239	1.593
Disease severity level	0.344 ± 0.018	0.328 ± 0.017	0.045	10.526
Defoliation	0.345 ± 0.018	0.293 ± 0.014	0.151	2.819
Exudates	0.343 ± 0.018	0.332 ± 0.017	0.032	15.248
Cankers	0.342 ± 0.020	0.327 ± 0.018	0.046	10.305

*Ht* represents total genetic diversity, *Hs* the mean within-population genetic diversity, *Gst* the genetic differentiation coefficients among different populations and *Nm* the gene flow number.

**Figure 2:**
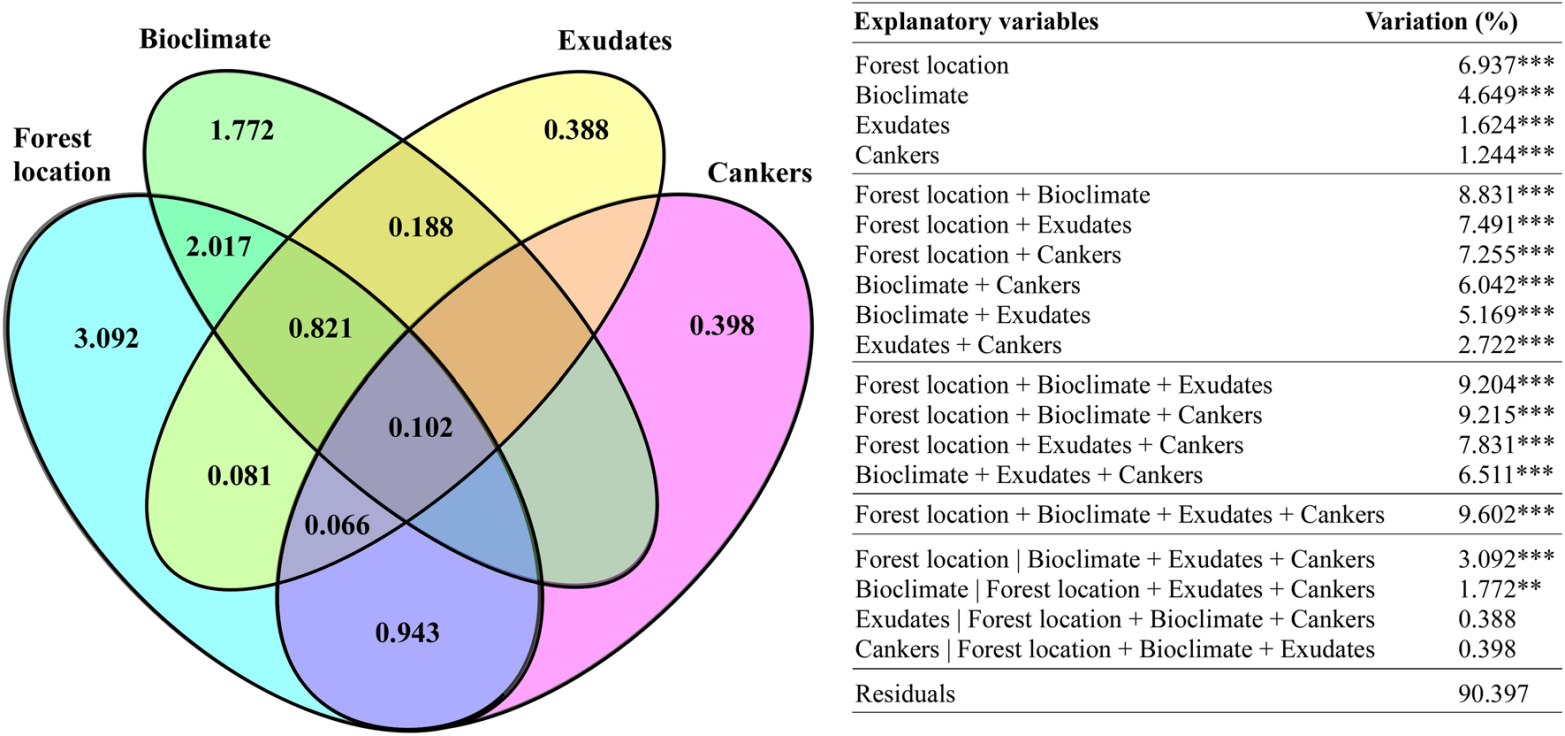
Contribution of the most significant variables for explaining genetic variability in *B.* *mediterranea* populations. The unique and shared contribution of variables is detailed. Percentage of variation was calculated by adjusted *R*^2^. ***represent *p* < 0.001 and **p < 0.01 for statistical significance of model.

In agreement with phylogenetic tree analysis from multilocus sequence analysis, principal coordinates analysis (PCoA) did not revealed clusters of cork oak isolates, according to the factors of interest (Fig. S1, both axes only capturing around 15% of data variation). Also, isolates from olive tree did not cluster all together and were closer to isolates from healthy and mild diseased trees. However, certain populations revealed a higher differentiation in allele frequencies than others, as evaluated by *Gst* coefficient (Table 2). There was a higher genetic differentiation among ‘forest’ (*Gst* = 0.239), followed by ‘defoliation’ (*Gst* = 0.151), and ‘bioclimate’ (*Gst* = 0.097) populations. The lowest genetic differentiation was found in ‘cankers’ (*Gst* = 0.046), ‘disease severity levels’ (*Gst* = 0.045) and ‘exudates’ (*Gst* = 0.032) populations. As expected, gene flow (*Nm*) values were opposite to *Gst*, being higher among populations with lower genetic differentiation and vice-versa (Table 2). These results agree with the genetic pairwise distances found in such populations (Table S1). Pairwise genetic distances in ‘forest’ achieved higher values (ranged from 0.244 to 0.067), than ‘defoliation’ (0.200 to 0.019), or bioclimate (0.085 to 0.053). Other disease-related variables (‘cankers’, ‘disease severity levels’ and ‘exudates’) never achieved more than 0.048 genetic distances. Among populations, isolates obtained from sub-humid bioclimate were the most genetically close from the ones obtained in the other forests (Table S1). Indeed, GR (a sub-humid forest) displayed the least genetic distance from all other forests. In contrast, isolates from humid bioclimate (in particular from AL forest) displayed the highest genetic distance from all other forests. Interestingly, isolates obtained from very accentuated defoliated trees revealed a high genetic distance from other isolates. AMOVA results revealed that variation within populations was always higher than among populations, which reinforces the high variability of *B.* *mediterranea* composition. In any case, variation among populations was higher for ‘forest location’ (10%), followed by ‘bioclimate’ (6%), ‘cankers’ (4%) and ‘exudates’ (3%), all at *p* < 0.001 (Table 3), suggesting higher genetic differentiation between regions and bioclimates. Accordingly, Mantel test shown significant correlation between *B.* *mediterranea* genetic diversity and geographic distance of cork oak forests (*R* = 0.105, *p* = 0.001). For further understanding the relative contribution of forest location, environmental, and disease-related factors in driving the genetic diversity of *B.* *mediterranea* populations, a redundancy analysis was performed (Fig. 2). The variables that explained the variation of *B.* *mediterranea* genetic diversity were ‘forest location’ and ‘bioclimate’ factors, followed by ‘exudates’ and ‘cankers’ (all at *p* < 0.001), being all the others (temperature, precipitation, disease severity levels, and defoliation) not significant. The combination of all significant variables explained 9.602% (*p* = 0.001) of *B.* *mediterranea* genetic variance. Most of this variation is due to the ‘bioclimate’ and ‘forest location’ factors that together contribute to 8.831% of variation (*p* = 0.001), in contrast with the shared contribution of ‘exudates’ and ‘cankers’ variables of only 2.722% of variation (*p* = 0.001). Indeed, when excluding all other variables, ‘exudates’ and ‘cankers’ do not present a significant contribution for *B.* *mediterranea* genetic diversity (*p* > 0.05). The production of exudates showed higher correlation with ‘bioclimate’ (0.188%) than with ‘forest location’ (0.081%). The opposite was found for the presence of cankers, in which no correlation was found with ‘bioclimate’, but a 0.943% correlation was found for ‘forest location’ variable. The index of association for the estimation of linkage disequilibrium revealed that LI and GV forests, as well as forests from the humid, sub-humid and semi-arid bioclimates was significantly different from 0, which indicates linkage disequilibrium (Table S2).

**Table 3:** Analysis of molecular variance (AMOVA) among and within *B.* *mediterranea* populations (Pops).

Source	df	SS	MS	Est. var	%	PhiPT
**Bioclimate**						0.062***
Among pops	3	75.087	25.029	0.815	6	
Within pops	61	748.328	12.268	12.268	94	
Total	64	823.415		13.083	100	
**Forest**						0.103***
Among pops	7	155.291	22.184	1.347	10	
Within pops	57	668.124	11.721	11.721	90	
Total	64	823.415		1.069	100	
**Disease severity level**						0.008
Among pops	2	29.700	14.850	0.105	1	
Within pops	62	793.715	12.802	12.802	99	
Total	64	823.415		12.907	100	
**Defoliation**						0.006
Among pops	4	54.603	13.651	0.076	1	
Within pops	60	768.813	12.814	12.814	99	
Total	64	823.415		12.889	100	
**Exudates**						0.032***
Among pops	1	26.030	26.030	0.414	3	
Within pops	63	797.386	12.657	12.657	97	
Total	64	823.415		13.071	100	
**Cankers**						0.040***
Among pops	1	22.945	22.945	0.523	4	
Within pops	63	800.470	12.706	12.706	96	
Total	64	823.415		13.229	100	

Asterisks on population differentiation (PhiPT) values represent significance of PhiPT values as ***p < 0.001.

df, degree of freedom; SS: sum of squares; MS: mean squares; Est. Var: estimated variance component; %: percentage of genetic variation.

## Discussion

In this study, the genetic diversity of endophytic *B. mediterranea* isolates was evaluated by multilocus phylogeny and microsatellite fingerprinting (MSP-PCR). The evaluated isolates comprised those obtained from cork oak trees, thriving in different forests, bioclimates, and displaying distinct charcoal disease severity levels and symptoms. This experimental approach is expected to provide new information about which factors/variables contribute the most to this species diversity and variability. Other isolates obtained from olive trees were also analyzed to evaluate host-specificity. As expected, all studied *B.* *mediterranea* isolates were phylogenetically apart from other *Biscogniauxia* species included in this study as outgroups, revealing the genetic divergence of *B.* *mediterranea* species. In general, individual (*i.e*., single-locus) and concatenated (*i.e.,* multi-locus) phylogenetic analyses did not correlate *B.* *mediterranea* isolates with any of the studied environmental/disease variables. Also, after determining the microsatellite polymorphic patterns, *B.* *mediterranea* isolates did not clustered according to the factors of interest. These results suggest a high genetic diversity and variability of *B.* *mediterranea* populations, which is concordant with other reports^9,15,17,18^, even when isolates come from the same stroma^16^. Furthermore, the inability to resolve *B.* *mediterranea* isolates obtained from cork oak and those obtained from olive tree reinforces current knowledge about the high adaptability of this fungus to different hosts, as suggested by^17^.

The genetic variability of *B.* *mediterranea* isolates was mostly explained by ‘forest location’ (6.9%) and ‘bioclimate’ (4.6%). Indeed, when these factors were combined, they explained 8.8% of *B.* *mediterranea* genetic variation. Considering ‘forest location’, *B.* *mediterranea* populations demonstrated the lowest genetic diversity within population, while also revealing the highest genetic differentiation and lowest gene flow among populations. These results suggest some isolation by distance of *B.* *mediterranea* communities, which agrees with the significant correlation (Mantel test) found between the *B.* *mediterranea* genetic diversity and forest geographic location of cork oak forests from which they were obtained. This contrasts with previous studies, where *B.* *mediterranea* intraspecific polymorphism and genetic diversity were not associated with geographic position of host trees^17^. However, the contribution of geographic isolation in *B.* *mediterranea* genetic differentiation was suggested for cork oak populations in Tunisia^18^. The high genetic variability of *B.* *mediterranea* has been related with the heterothallic mating system of this species^15^ and sexual reproduction with the production of a high number of variable ascospores^21^. Our results suggest that global *B. mediterranea* population displays linkage disequilibrium and a clonal genetic structure. Despite that, populations from the majority of forests (PG-ER, PG-RC, AL, GR, HC-CT and HC-MA) demonstrated to have random mating with frequent sexual reproduction. *B.* *mediterranea* ascospores are primarily dispersed by wind after the occurrence of precipitation^21,22^, although insects could also play a role for their spreading in short- and long- distances, depending on their bioecology^21,22^. Our results suggest low migration rates between geographically distant *B.* *mediterranea* populations, indicating short-distance dispersal. The clonal structure of the populations from the majority of bioclimates and the finding that PG-ER and AL forests presented the lowest genetic diversity among all other sampled forests reinforces this suggestion. In contrast with other sampled forests, both are characterized by a high density of mixed forest trees and low anthropogenic interference, which may restrain spore dispersal to and from distant locations. Indeed, canopy architecture and the use of mixed tree species have been reported as a management strategy to reduce spore dispersal of pathogenic fungi^23–25^. In addition, high genetic differentiation among forest populations can be a result of random events but there is enough gene flow to refute the effects of genetic drift^26^. While a significant variation was found among forest populations, high variability of *B.* *mediterranea* was also described within populations. This result concurs with the high intraspecific genetic variability found within populations described by Henriques et al*.*^27^, indicating an adaptation of *B.* *mediterranea* to the environment and ensuring species long-term survival.

Besides ‘forest location’ factor, a significant variation on *B.* *mediterranea* populations occurred in response to ‘bioclimate’, although precipitation and temperature alone were not significantly correlated with *B.* *mediterranea* variability, as revealed by variation partitioning redundancy analysis. In contrast, other studies indicated a positive correlation between *B.* *mediterranea* genetic diversity with temperature and rainfall^18^. The ability of *B.* *mediterranea* to develop in a wide range of temperatures^27^, associated with the significant variability of this fungus in different bioclimates may represent a problem for charcoal cork oak disease management. This will be further challenged by the effect of combined bioclimate and charcoal symptoms (exudates in cork oak trunk) in increasing *B.* *mediterranea* variability. Indeed, in Tunisia, the correlation between bioclimate and charcoal disease development has been suggested^7^. In any case, the variability promoted by disease symptoms (exudates and cankers presence) was better explained when taking into consideration the forest location, suggesting that other factors characteristic of each forest (*i.e*., silvicultural practices not included in this study) are contributing to *B. mediterranea* variability. Moreover, *B.* *mediterranea* isolates collected from declining trees or trees with charcoal symptoms, like trunk exudates, presented a higher genetic diversity than those collected from healthier trees.

## Conclusions

Several reports suggested that *Quercus suber* populations and their associated microbiota are vulnerable to different bioclimates^28–30^ and will be affected by the predicted climate changes^31^. Therefore, as cork oak forests currently displaying a moist bioclimate become more arid, they will be increasingly affected by environmental stressors. Taking all together, the results obtained in this work support the previous suggestions that *B.* *mediterranea* isolates have facilitated adaptation. Our findings reinforce the previous knowledge of *B. mediterranea* opportunistic behavior and reveal the importance of bioclimate as a source of *B.* *mediterranea* variability, exacerbating the implications of a changing climate on cork oak forests sustainability which will affect cork production and quality.

## Methods

### *Biscogniauxia mediterranea* isolates

**Figure 3:**
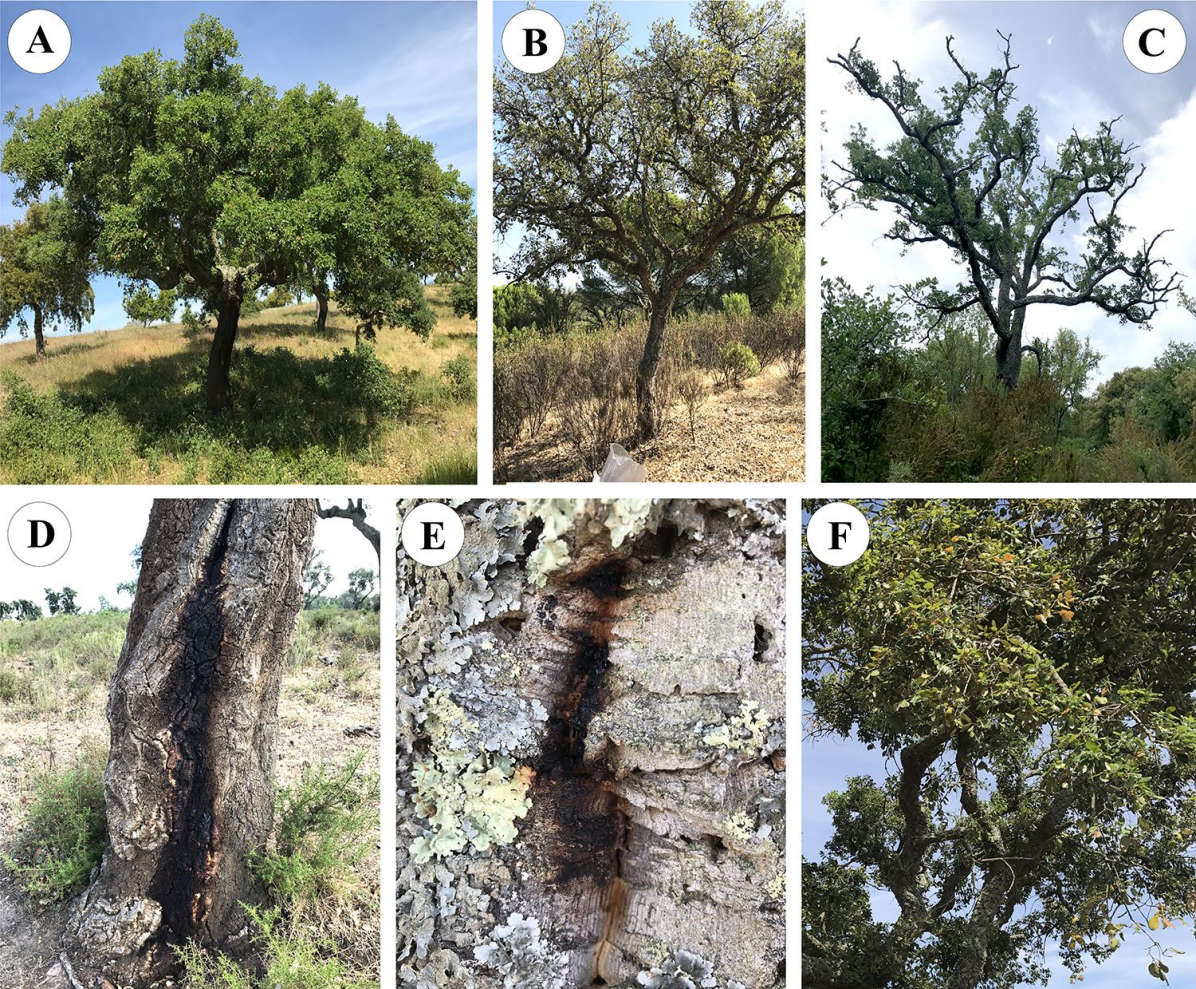
Charcoal disease severity levels (**A**–**C**) and disease symptoms (**D**,**E**), displayed by sampled cork oak trees. (**A**) Healthy tree; (**B**) Tree with mild symptoms; (**C**) declining tree; (**D**) presence of canker and exudates; (**E**) presence of exudates on uncorked tree; (**F**) presence of decolorated and dried leaves.

*Biscogniauxia mediterranea* isolates were obtained from cork oak twigs as endophytes^32^. Twigs were sampled, during 2017, from eight Portuguese cork oak forests with different locations and bioclimate classifications (Fig. S2; Table S3). Bioclimate for each forest was determined using Emberger index and Emberger climatogram^33,34^ and ranged from hyper-humid to semi-arid bioclimates [hyper-humid (PG-RC and PG-ER), humid (AL and LI), sub-humid (GV and GR) and semi-arid (HC-MA and HC-CT)]. If existent, trees with different disease severity levels were collected from each location. Evaluated disease symptoms included defoliation (5 levels: 0–10%—no damage; 11–25%—light damage; 26–50%—moderate damage; 51–90%—severe damage; > 90%—extreme damage), as well as canopy and trunk damages (3 levels: 0—no damage; 1—moderate damage; 2—severe damage) (Table S4). Disease damages included dried, wilting and decolorated leaves, presence of cankers, decolorated trunk and presence of exudates (Fig. 3). Disease severity levels were grouped into three categories (healthy, mild, and declining), considering the combination of different symptoms and corresponding levels, as described elsewhere^32^. Endophytic isolates were obtained by sterilizing the surface of cork oak twigs, plating twigs onto Potato Dextrose Agar (PDA) medium and obtaining pure cultures through re-plating outgrowing fungi into fresh PDA medium^30^. DNA of pure cultures was extracted using Quick-DNA Fungal/Bacterial Miniprep Kit (Zymo Research, Irvine, CA, USA) and *B.* *mediterranea* isolates were identified using universal primer pairs *ITS1-F* (5′-CTTGGTCATTTAGAGGAAGTAA-3′) and *ITS4* (5′-TCCTCCGCTTATTGATATGC-3′)^35^. A total of 74 *B.* *mediterranea* isolates obtained from cork oak were distinguished and identified by sequencing (Table 4). Three other isolates were obtained from *Olea europaea* twigs (Bm78^36^) and olives (Bm79 and Bm80^37^), collected from cvs. *Cobrançosa* (Bm78 and Bm80) and *Madural* (Bm79). These *O. europaea*-derived isolates were included in this study to evaluate host-specificity in *B. mediterranea*. All methods complied with relevant institutional, national, and international guidelines and legislation.

**Table 4:** GenBank accession numbers for each sequenced locus [internal transcribed spacer (*ITS*), translation elongation factor 1-α (*TEF*), partial glutamine synthetase (*GS*), actin (*ACT*), chitin synthase 1 (*CHS*) and β-tubulin 2 (*TUB2*)] of endophytic *B. mediterranea* isolates obtained from cork oak twigs and other fungi used in this study.

Isolate	Location	Forest	Tree number	Bioclimate	Disease severity level	GenBank accession numbers					
						*ITS*	*TEF*	*GS*	*ACT*	*CHS*	*TUB2*
Bm02	Alcobaça	AL	4	Humid	Mild	MZ502502	MZ713533	–	MZ700363	MZ700468	MZ713420
Bm03^a^	Alcobaça	AL	4	Humid	Mild	MZ502503	MZ713531	MZ713581	MZ700389	MZ700426	MZ713421
Bm04	Alcobaça	AL	2	Humid	Healthy	–	MZ713498	MZ713613	MZ700402	–	MZ713459
Bm05^a^	Alcobaça	AL	2	Humid	Healthy	MZ502504	MZ713498	MZ713565	MZ700418	MZ700473	MZ713430
Bm06	Alcobaça	AL	2	Humid	Healthy	MZ502505	MZ713499	–	–	MZ700472	–
Bm07^a^	Limãos	LI	5	Humid	Mild	MZ502506	MZ713490	MZ713608	MZ700403	MZ700441	MZ713484
Bm08^a^	Limãos	LI	2	Humid	Declining	MZ502507	MZ713496	MZ713599	MZ700365	MZ700432	MZ713438
Bm09^a^	Limãos	LI	3	Humid	Mild	MZ502508	MZ713543	MZ713588	MZ700374	MZ700440	MZ713460
Bm10^a^	Limãos	LI	1	Humid	Mild	MZ502509	MZ713500	MZ713579	MZ700381	MZ700482	MZ713427
Bm11^a^	Limãos	LI	3	Humid	Mild	MZ502510	MZ713521	MZ713572	MZ700392	MZ700442	MZ713486
Bm12^a^	Limãos	LI	1	Humid	Mild	MZ502511	MZ713520	MZ713569	MZ700412	MZ700443	MZ713461
Bm13	Limãos	LI	2	Humid	Declining	MZ502512	MZ713491	MZ713574	MZ700413	–	MZ713462
Bm14^a^	Limãos	LI	1	Humid	Mild	MZ502513	MZ713493	MZ713609	MZ700411	MZ700444	MZ713425
Bm15^a^	Limãos	LI	3	Humid	Mild	MZ502514	MZ713528	MZ713568	MZ700423	MZ700430	MZ713463
Bm16^a^	Limãos	LI	1	Humid	Mild	MZ502515	MZ713501	MZ713607	MZ700371	MZ700431	MZ713483
Bm17^a^	Limãos	LI	1	Humid	Mild	MZ502516	MZ713549	MZ713567	MZ700364	MZ700492	MZ713431
Bm18^a^	Limãos	LI	5	Humid	Mild	MZ502517	MZ713502	MZ713614	MZ700404	MZ700491	MZ713448
Bm19^a^	Limãos	LI	3	Humid	Mild	MZ502518	MZ713503	MZ713606	MZ700393	MZ700481	MZ713449
Bm20^a^	Limãos	LI	1	Humid	Mild	MZ502519	MZ713492	MZ713603	MZ700414	MZ700460	MZ713457
Bm21	Limãos	LI	1	Humid	Mild	MZ502520	MZ713529	MZ713577	–	MZ700483	MZ713464
Bm22^a^	Limãos	LI	6	Humid	Mild	MZ502521	MZ713504	MZ713621	MZ700420	MZ700477	MZ713465
Bm23^a^	Herdade Contenda	HC-MA	4	Semi-arid	Declining	MZ502522	MZ713550	MZ713622	MZ700383	MZ700471	MZ713487
Bm24^a^	Herdade Contenda	HC-MA	6	Semi-arid	Healthy	MZ502523	MZ713551	MZ713585	MZ700410	MZ700429	MZ713456
Bm25^a^	Herdade Contenda	HC-MA	6	Semi-arid	Healthy	MZ502524	MZ713544	MZ713619	MZ700382	MZ700437	MZ713473
Bm26	Herdade Contenda	HC-MA	5	Semi-arid	Mild	MZ502525	MZ713552	MZ713583	MZ700394	MZ700484	MZ713426
Bm27^a^	Herdade Contenda	HC-MA	5	Semi-arid	Mild	MZ502526	MZ713505	MZ713600	MZ700370	MZ700480	MZ713439
Bm28	Herdade Contenda	HC-MA	6	Semi-arid	Healthy	MZ502527	MZ713506	MZ713595	–	MZ700490	MZ713475
Bm29^a^	Herdade Contenda	HC-MA	2	Semi-arid	Mild	MZ502528	MZ713489	MZ713612	MZ700359	MZ700485	MZ713450
Bm30^a^	Herdade Contenda	HC-MA	2	Semi-arid	Mild	MZ502529	MZ713524	MZ713616	MZ700415	MZ700489	MZ713447
Bm31	Peneda Gerês	PG-RC	4	Hyper-humid	Healthy	MZ502530	MZ713527	MZ713584	–	MZ700461	MZ713437
Bm32	Peneda Gerês	PG-RC	3	Hyper-humid	Healthy	MZ502531	MZ713548	MZ713605	–	MZ700427	MZ713476
Bm33	Peneda Gerês	PG-RC	4	Hyper-humid	Healthy	MZ502532	MZ713534	MZ713570	–	MZ700445	MZ713455
Bm34	Peneda Gerês	PG-RC	2	Hyper-humid	Healthy	MZ502533	MZ713507	–	MZ700405	MZ700479	MZ713466
Bm35	Peneda Gerês	PG-RC	1	Hyper-humid	Mild	MZ502534	–	–	MZ700419	MZ700446	MZ713482
Bm36^a^	Peneda Gerês	PG-RC	5	Hyper-humid	Healthy	MZ502535	MZ713508	MZ713586	MZ700362	MZ700459	MZ713440
Bm37^a^	Peneda Gerês	PG-RC	2	Hyper-humid	Healthy	MZ502536	MZ713518	MZ713604	MZ700378	MZ700476	MZ713467
Bm38	Peneda Gerês	PG-RC	2	Hyper-humid	Healthy	MZ502537	MZ713497	MZ713620	MZ700375	–	MZ713422
Bm39^a^	Peneda Gerês	PG-ER	4	Hyper-humid	Healthy	MZ502538	MZ713509	–	MZ700417	MZ700447	MZ713446
Bm41^a^	Peneda Gerês	PG-ER	4	Hyper-humid	Healthy	MZ502539	MZ713545	MZ713587	MZ700406	MZ700475	MZ713436
Bm42^a^	Peneda Gerês	PG-ER	3	Hyper-humid	Mild	MZ502540	MZ713542	MZ713617	MZ700407	MZ700448	MZ713468
Bm43^a^	Grândola	GR	1	Sub-humid	Declining	MZ502541	MZ713553	MZ713602	MZ700395	MZ700462	MZ713469
Bm44^a^	Grândola	GR	2	Sub-humid	Declining	MZ502542	MZ713516	MZ713591	MZ700401	MZ700474	MZ713419
Bm45	Grândola	GR	4	Sub-humid	Mild	MZ502543	MZ713557	–	MZ700369	MZ700463	MZ713445
Bm46^a^	Grândola	GR	3	Sub-humid	Mild	MZ502544	MZ713515	MZ713590	MZ700400	MZ700458	MZ713454
Bm47^a^	Grândola	GR	4	Sub-humid	Mild	MZ502545	MZ713535	MZ713558	MZ700408	MZ700449	MZ713451
Bm48^a^	Grândola	GR	4	Sub-humid	Mild	MZ502546	MZ713522	MZ713623	MZ700399	MZ700467	MZ713474
Bm49^a^	Grândola	GR	2	Sub-humid	Declining	MZ502547	MZ713556	MZ713560	MZ700409	MZ700457	MZ713453
Bm50^a^	Grândola	GR	1	Sub-humid	Declining	MZ502548	MZ713555	MZ713571	MZ700372	MZ700488	MZ713481
Bm51	Grândola	GR	3	Sub-humid	Mild	MZ502549	MZ713526	–	MZ700361	MZ700466	MZ713432
Bm52^a^	Grândola	GR	1	Sub-humid	Declining	MZ502550	MZ713514	MZ713561	MZ700376	MZ700464	MZ713477
Bm53	Grândola	GR	5	Sub-humid	Declining	MZ502551	MZ713536	MZ713559	–	MZ700470	MZ713423
Bm54^a^	Grândola	GR	1	Sub-humid	Declining	MZ502552	MZ713510	MZ713578	MZ700366	MZ700450	MZ713480
Bm55^a^	Grândola	GR	4	Sub-humid	Mild	MZ502553	MZ713541	MZ713575	MZ700386	MZ700456	MZ713485
Bm56^a^	Grândola	GR	3	Sub-humid	Mild	MZ502554	MZ713511	MZ713596	MZ700387	MZ700439	MZ713479
Bm57^a^	Grândola	GR	2	Sub-humid	Declining	MZ502555	MZ713523	MZ713611	MZ700377	MZ700436	MZ713418
Bm58^a^	Grândola	GR	3	Sub-humid	Mild	MZ502556	MZ713495	MZ713593	MZ700416	MZ700487	MZ713444
Bm59	Grândola	GR	1	Sub-humid	Declining	MZ502557	–	–	–	–	–
Bm60^a^	Gavião	GV	1	Sub-humid	Declining	MZ502558	MZ713530	MZ713576	MZ700360	MZ700438	MZ713433
Bm61^a^	Gavião	GV	1	Sub-humid	Declining	MZ502559	MZ713537	MZ713573	MZ700379	MZ700469	MZ713441
Bm62	Gavião	GV	3	Sub-humid	Mild	–	–	MZ713610	MZ700421	–	MZ713443
Bm63^a^	Gavião	GV	1	Sub-humid	Declining	MZ502560	MZ713519	MZ713601	MZ700398	MZ700451	MZ713458
Bm64^a^	Gavião	GV	2	Sub-humid	Declining	MZ502561	MZ713554	MZ713589	MZ700390	MZ700455	MZ713452
Bm65	Gavião	GV	4	Sub-humid	Mild	MZ502562	–	MZ713563	MZ700358	MZ700425	–
Bm66^a^	Gavião	GV	4	Sub-humid	Mild	MZ502563	MZ713513	MZ713615	MZ700368	MZ700465	MZ713434
Bm67^a^	Gavião	GV	1	Sub-humid	Declining	MZ502564	MZ713547	MZ713562	MZ700385	MZ700424	MZ713417
Bm68^a^	Herdade Contenda	HC-CT	5	Semi-arid	Mild	MZ502565	MZ713538	MZ713566	MZ700396	MZ700435	MZ713470
Bm69^a^	Herdade Contenda	HC-CT	6	Semi-arid	Declining	MZ502566	MZ713525	MZ713580	MZ700367	MZ700428	MZ713429
Bm70	Herdade Contenda	HC-CT	2	Semi-arid	Declining	MZ502567	–	MZ713564	–	–	–
Bm71	Herdade Contenda	HC-CT	5	Semi-arid	Mild	MZ502568	MZ713540	–	–	–	–
Bm72^a^	Herdade Contenda	HC-CT	5	Semi-arid	Mild	MZ502569	MZ713512	MZ713625	MZ700422	MZ700452	MZ713428
Bm73	Herdade Contenda	HC-CT	5	Semi-arid	Mild	MZ502570	–	MZ713624	MZ700384	MZ700434	MZ713424
Bm74	Herdade Contenda	HC-CT	5	Semi-arid	Mild	MZ502571	–	MZ713592	MZ700388	-	MZ713478
Bm75^a^	Herdade Contenda	HC-CT	2	Semi-arid	Declining	MZ502572	MZ713488	MZ713598	MZ700391	MZ700454	MZ713472
Bm76	Herdade Contenda	HC-CT	3	Semi-arid	Mild	MZ502573	MZ713546	MZ713582	–	MZ700478	MZ713471
Bm78^b^	Mirandela	–	–	–	–	MZ502574	MZ713539	MZ713594	MZ700380	MZ700433	–
Bm79^b^	Mirandela	–	–	–	–	MZ502575	MZ713494	MZ713618	MZ700373	MZ700453	MZ713442
Bm80^b^	Mirandela	–	–	–	–	MZ502576	MZ713532	MZ713597	MZ700397	MZ700486	MZ713435
*B. atropunctata*	–	–	–	–	–	–	JX507799	–	AY951785	AF200442	–
*B. nummularia*	–	–	–	–	–	–	MH860015	Scaffold_43: 11,007–11,944	GQ428312	AF200443	KX271241
*Xylaria hypoxylon*	–	–	–	–	–	AY327490	AM993138	–	AY327483	AF200448	KX271279

Information is given concerning the sampled forests and disease severity level of sampled host trees. Isolates without GenBank accession number were only used for microsatellite-primed PCR fingerprinting.

^a^Refers to sequences used for multilocus analysis.

^b^Refers to *B.* *mediterranea* specimens obtained from olive trees.

### Multilocus sequence analysis

Multilocus phylogenetic analysis was performed by targeting several loci: internal transcribed spacer (*ITS*), translation elongation factor 1-α (*TEF*), partial glutamine synthetase (*GS*), actin (*ACT*), chitin synthase 1 (*CHS*) and β-tubulin 2 (*TUB2*). PCR amplifications were performed according to each pair of primers and target region (Table 5). PCR reactions were prepared for all loci in 10 µl volume using 0.2 U/µl of NZYTaq II 2× Green Master Mix (NZYTech, Portugal), 0.5 µM of each primer and 1 µl of DNA. PCR products were run on a 1% (w/v) agarose gel, stained with Green Safe Premium (NZYTech, Portugal). Isopropanol 75% (v/v) was used to purify PCR products and sequencing was performed by Macrogen, Inc services (Madrid, Spain).

**Table 5:** Locus regions amplified for *B. mediterranea* phylogenetic analyses and correspondent PCR conditions. The corresponding amplicons size are shown in brackets.

Locus	Primers	PCR program	Refs.
*ITS* (600 bp)	*ITS1-F* 5′-CTTGGTCATTTAGAGGAAGTAA *ITS4* 5′-TCCTCCGCTTATTGATATGC	*Initial denaturation*: 94 °C for 5 min 35 *cycles*: 30 s at 94 °C; 30 s at 54 °C; 60 s at 72 °C *Final elongation*: 72 °C for 10 min	^35^
*TEF* (350 bp)	*EF 1-728 F* 5′-CATCGAGAAGTTCGAGAAGG *EF 1-986 R* 5′-TACTTGAAGGAACCCTTACC	*Initial denaturation*: 96 °C for 3 min 40 *cycles*: 30 s at 95 °C; 45 s at 54 °C; 45 s at 72 °C *Final elongation*: 72 °C for 7 min	^57^
*GS* (700 bp)	*GSF1* 5′-ATGGCCGAGTACATCTGG *GSR1* 5′-GAACCGTCGAAGTTCCAG	*Initial denaturation*: 95 °C for 4 min 35 *cycles*: 30 s at 95 °C; 30 s at 54 °C; 45 s at 72 °C *Final elongation*: 72 °C for 7 min	^58^
*ACT* (900 bp)	*ACT-1* 5′-TGGGACGATATGGAIAAIATCTGGCA *ACT-4R* 5′-TCITCGTATTCTTGCTTIGAIATCCACAT	*Initial denaturation*: 94 °C for 5 min 35 *cycles*: 30 s at 95 °C; 30 s at 57 °C; 60 s at 72 °C *Final elongation*: 72 °C for 7 min	^59^
*CHS* (300 bp)	*CHS-79 F* 5′-TGGGGCAAGGATGCTTGGAAGAAG *CHS-354 R* 5′-TGGAAGAACCATCTGTGAGAGTTG	*Initial denaturation*: 95 °C for 2 min 40 *cycles*: 60 s at 95 °C; 30 s at 62 °C; 20 s at 72 °C *Final elongation*: 72 °C for 5 min	^57^
*TUB2* (500 bp)	*Bt2a* 5′-GGTAACCAAATCGGTGCTGCTTTC *Bt2b* 5′-ACCCTCAGTGTAGTGACCCTTGGC	*Initial denaturation*: 95 °C for 5 min 35 *cycles*: 30 s at 95 °C; 30 s at 58 °C; 60 s at 72 °C *Final elongation*: 72 °C for 7 min	^60^

DNA sequences were processed using AB1 trace files in Geneious version 2010.4.8.5 (Biomatters, New Zealand), unless stated otherwise. For each isolate and molecular marker, forward and reverse sequences were trimmed (0.05 error probability limit), assembled and consensus sequences were created. Consensus sequences obtained in this study were deposited in GenBank (for accession numbers, see Table 4). Multiple sequence alignments of each region were performed by using MUSCLE version 3.5 algorithm with a maximum of 10 iterations. Distance measure used for 1st iteration was kmer4_6 and subsequent iterations were run with pctid_kimura. All iterations were performed using UPGMB clustering method and CLUSTALW sequence weighting scheme. If needed, alignments were manually edited and Gblocks (web-based, version 0.91b, January 2002, http://molevol.cmima.csic.es/castresana/Gblocks_server.html, last accessed date: 08/02/2021) was used to eliminate poorly aligned positions and divergent regions, allowing smaller final blocks^38^. Geneious version 2010.4.8.5 was then used to concatenate alignments. Some isolates were not sequenced with enough quality in some targeted loci and were excluded from individual alignments before concatenation. DNA sequences from closely related taxa—*Biscogniauxia atropunctata*, *B.* *nummularia* and *Xylaria hypoxylon*^39,40^—were used as outgroups and were retrieved from GenBank (Table 4).

Phylogenetic trees for each individual locus were generated using sequences from 74 *B. mediterranea* isolates for *ITS*, 70 for *TEF*, 68 for *GS*, 66 for *ACT*, 69 for *CHS* and 71 for *TUB2* (Table 4). The final concatenated alignment used to build the multilocus phylogenetic tree included sequences from 52 *B.* *mediterranea* isolates and from the three outgroup species (isolates marked with * in Table 4). PartitionFinder2 version 2.1.1 was run on CIPRES Science Gateway (web-based, version 3.3, https://www.phylo.org/portal2/, last accessed date: 17/02/2021)^41^ to find best-fit partition schemes of each loci^42^. Bayesian inference (BI) trees were computed using Markov chain Monte Carlo (MCMC) algorithm in MrBayes version 3.2.7^43^. Models (lset) and prior probability distributions (prset) were set according to PartitionFinder2 results. Two independent runs were performed with one million generations and four chains in each run. The two runs were converged with a burnin of 25% and tree with posterior probabilities was generated. Maximum Likelihood (ML) trees were generated using W-IQ-TREE (http://iqtree.cibiv.univie.ac.at/, last accessed date: 17/02/2021)^44^, a web server for IQ-TREE^45^. Best-fit model was computed using ModelFinder version 1.4.2^46^, with an edge-linked partition model^47^. Branch support analysis was performed using 1000 ultrafast bootstrap replicates^48^ and minimum correlation coefficient of 0.99. Phylogenetics trees were visualized in FigTree version 1.4.4^49^. ML bootstrap (BS) and BI posterior probability (PP) values and topologies obtained by both phylogenetic inference methods were compared using TreeGraph 2 version 2.0.40^50^. This allowed the distinction of clades with reliable support value^19,20^ or groups of sequences showing limited support. Additional data from environmental/disease factors of interest were added using the vector image editor Inkscape version 0.92^51^.

### Microsatellite-primed PCR fingerprinting

The molecular diversity of 68 *B.* *mediterranea* isolates was also evaluated by using microsatellite-primed PCR (MSP-PCR) with four sets of primers, *(GTG)*_*5*_, *(CAG)*_*5*_, *(ACAC)*_*5*_ and *M13* (phage M13 core sequence; 5′-GAGGGTGGNGGNTCT)^17,18^. PCR reactions were prepared in 10 µl volume using 0.2 U/µl of NZYTaq II 2× Green Master Mix (NZYTech, Portugal), 1 µM of each primer and 1 µl of DNA. The amplifications with *(GTG)*_*5*_ were performed using the following PCR program: initial denaturation at 94 °C, for 4 min; 35 cycles of 45 s at 94 °C, 45 s at 56 °C and 30 s at 72 °C; and final elongation at 72 °C for 10 min. Amplifications with *(CAG)*_*5*_, *(ACAC)*_*5*_ and *M13* primers comprised the following PCR program: initial denaturation at 94 °C, for 2 min; 40 cycles of 30 s at 93 °C, 60 s at 53 °C and 30 s at 72 °C; and final elongation at 72 °C for 10 min. Amplifications with each primer were performed in duplicate for reproducibility. PCR products were run on a 1.5% (w/v) agarose gel, stained with Green Safe Premium (NZYTech, Portugal). The visualization and image acquisition were performed using an UV transilluminator (VWR Genosmart, United Kingdom). DNA fingerprinting gel images were analyzed using GelAnalyzer version 19.1 (http://www.gelanalyzer.com) and each band was scored as 0 (absence) or 1 (presence).

Molecular diversity of *B.* *mediterranea* isolates was evaluated by grouping samples into populations, based on their host origin, such as climate parameters (including bioclimate), forest provenience, or disease-related parameters (including severity level, defoliation, and presence of exudates). Frequency- and distance-based genetic diversity of each population was evaluated using GenAlEX version 6.51b2^52^. Frequency-based genetic diversity was evaluated considering the number of different alleles (*Na*), number of effective alleles (*Ne*), Shannon’s Information index (*I*), diversity [*h* = 1 − (p^2^ + q^2^)] and percentage of polymorphic loci (PPL). Distance-based genetic diversity was accessed using different analyses: Principal Coordinates Analysis (PCoA) to find patterns within dataset; Nei’s pairwise genetic distance to calculate genetic distance between populations; Analysis of Molecular Variance (AMOVA) to calculate hierarchical partitioning of genetic variation within and among populations (999 permutations); and Mantel test to calculate statistical correlation between genetic diversity and geographic distance (999 permutations). *F*-statistics to analyze diversity and genetic differentiation among populations were calculated in POPGENE version 1.32^53^. Given the reduced sample size, *B.* *mediterranea* isolates collected from olive tree (Bm78, Bm79 and Bm80) were included only in phylogenetic and PCoA analyses.

Redundancy analysis (RDA) was used to explore responses of *B.* *mediterranea* composition to environmental (bioclimate, mean maximum and minimum temperatures and mean total precipitation for the 10 years previous to sampling collection) and disease variables (disease severity level, exudates, cankers, and defoliation), by making use of the R version 4.0.2^54^. Analyses were performed using the package *vegan* version 2.5-7^55^, except when stated otherwise. Spatial trend was included in RDA using a trend surface analysis. Latitude–longitude data was transformed into flat Cartesian coordinates using *geoXY()* of SoDA package (version 1.0-6.1)^56^. To compute polynomials of degree 3, *poly()* of STATS package (version 4.0.2) was used. Spatial and environmental variables were analyzed separately on a first approach. RDA was performed using *rda()*, while *anova.cca()* was used to perform Monte Carlo permutation test (1000 permutations) and test significance of global model. Forward selection of explanatory variables was performed using *ordistep()* with 999 permutations, using spatial and environmental variables to find most parsimonious RDA. Then, another RDA was performed, as described before, for selected variables. The contribution to variation of those variables was performed using *RsquareAdj()* and Monte Carlo permutation test (1000 permutations) to determine significance. For explaining the variation on *B.* *mediterranea* composition, variation partitioning was performed for the best model, using the most explanatory variables (combination of spatial and environmental variables), and making use of *varpart()*. Partial-RDA was performed to evaluate the influence of conditional variables obtained from variation partition. Statistical significance was performed as described for RDA. Spatial variable was referred as ‘forest location’ variable in sections related with this analysis. Linkage disequilibrium of *B. mediterranea* populations was evaluated by the index of association (I_A_) and the unbiased index of association ($\overline{r}$_d_), in which clonal populations have results significantly different from 0 and sexual populations do not have statistical significant I_A_ and $\overline{r}$_d_^61^. Analysis was performed using *popsub()* to specify population and *ia()* with 999 permutations for calculation using poppr package (version 2.9.0)^62^.

## Supplementary Information

Supplementary Information.

## References

[1] FAO and Plan Bleu. *State of Mediterranean Forests 2018* (Food and Agriculture Organization of the United Nations, 2018).

[2] Gauquelin T (2018). Mediterranean forests, land use and climate change: A social-ecological perspective. Reg. Environ. Change..

[3] Oliveira V, Lauw A, Pereira H (2016). Sensitivity of cork growth to drought events: Insights from a 24-year chronology. Clim. Change.

[4] Acácio V, Dias FS, Catry FX, Rocha M, Moreira F (2017). Landscape dynamics in Mediterranean oak forests under global change: Understanding the role of anthropogenic and environmental drivers across forest types. Glob. Change Biol..

[5] Moricca S (2016). Endemic and emerging pathogens threatening cork oak trees: Management options for conserving a unique forest ecosystem. Plant Dis..

[6] Touhami I (2020). Decline and dieback of cork oak (*Quercus suber* L.) forests in the Mediterranean basin: A case study of Kroumirie, Northwest Tunisia. J. For. Res..

[7] Yangui I (2021). Occurrence of *Biscogniauxia**mediterranea* in cork oak stands in Tunisia. Phytoparasitica.

[8] Linaldeddu BT, Sirca C, Spano D, Franceschini A (2011). Variation of endophytic cork oak-associated fungal communities in relation to plant health and water stress. For. Pathol..

[9] Schiaffino, A., Franceschini, A., Maddau, L. & Serra, S. Molecular characterisation of *Biscogniauxia mediterranea* (De Not.) O. Kuntze strains isolated from declining trees of *Quercus suber* L. in Sardinia. In *Protection intégrée des forêts de chênes*, Vol 27, 26–29 (2001).

[10] Linaldeddu BT, Franceschini A, Pulina MA (2005). Epidemiological aspects of *Biscogniauxia mediterranea* in declining cork oak forest in Sardinia (Italy). IOBC/WPRS Bull..

[11] Linaldeddu BT, Sirca C, Spano D, Franceschini A (2009). Physiological responses of cork oak and holm oak to infection by fungal pathogens involved in oak decline. For. Pathol..

[12] Henriques J, Inácio ML, Lima A, Sousa E (2012). New outbreaks of charcoal canker on young cork oak trees in Portugal. IOBC/wprs Bull..

[13] Nugent LK, Sihanonth P, Thienhirun S, Whalley AJS (2005). *Biscogniauxia*: A genus of latent invaders. Mycologist.

[14] Giraud T, Enjalbert J, Fournier E, Delmotte F, Dutech C (2008). Population genetics of fungal diseases of plants. Parasite.

[15] Vannini A, Mazzaglia A, Anselmi N (1999). Use of random amplified polymorphic DNA (RAPD) for detection of genetic variation and proof of the heterothallic mating system in *Hypoxylon mediterraneum*. Eur. J. For. Pathol..

[16] Henriques J, Nóbrega F, Sousa E, Lima A (2014). Diversity of *Biscogniauxia mediterranea* within single stromata on cork oak. J. Mycol..

[17] Henriques J, Nóbrega F, Sousa E, Lima A (2016). Analysis of the genetic diversity and phylogenetic relationships of *Biscogniauxia mediterranea* isolates associated with cork oak. Phytoparasitica.

[18] Yangui I (2019). *Biscogniauxia mediterranea* associated with cork oak (*Quercus **suber*) in Tunisia: Relationships between phenotypic variation, genetic diversity and ecological factors. Fungal Ecol..

[19] Erixon P, Svennblad B, Britton T, Oxelman B (2003). Reliability of bayesian posterior probabilities and bootstrap frequencies in phylogenetics. Syst. Biol..

[20] Hillis DM, Bull JJ (1993). An empirical test of bootstrapping as a method for assessing confidence in phylogenetic analysis. Syst. Biol..

[21] Jiménez J, Trapero Casas AE (2005). Chancro carbonoso de ‘*Quercus*’ III: dispersión de ascosporas del agente casual. Boletín Sanid. Veg. Plagas.

[22] Henriques J (2014). Factors affecting the dispersion of *Biscogniauxia mediterranea* in Portuguese cork oak stands. Silva Lusit..

[23] Field E (2020). Associational resistance to both insect and pathogen damage in mixed forests is modulated by tree neighbour identity and drought. J. Ecol..

[24] Roberts M (2020). The effect of forest management options on forest resilience to pathogens. Front. For. Glob. Chang..

[25] Vidal T (2017). Reduction of fungal disease spread in cultivar mixtures: Impact of canopy architecture on rain-splash dispersal and on crop microclimate. Agric. For. Meteorol..

[26] Wright S (1931). Evolution in Mendelian populations. Genetics.

[27] Henriques J (2015). Morphological and genetic diversity of *Biscogniauxia mediterranea* associated to *Quercus **suber* in the Mediterranean basin. Rev. Ciências Agrárias.

[28] Reis F (2018). Ectomycorrhizal fungal diversity and community structure associated with cork oak in different landscapes. Mycorrhiza.

[29] Reis F (2019). Climatic impacts on the bacterial community profiles of cork oak soils. Appl. Soil Ecol..

[30] Costa D, Tavares R, Baptista P, Lino-Neto T (2018). Diversity of fungal endophytic community in *Quercus suber* L. under different climate scenarios. Rev. Ciências Agrárias.

[31] Vanhove, M. *et al.* Using gradient forest to predict climate response and adaptation in cork oak. *J. Evol. Biol.* **34**, 910–923 (2021). 10.1111/jeb.13765 33484040

[32] Costa D, Tavares RM, Baptista P, Lino-Neto T (2020). Cork oak endophytic fungi as potential biocontrol agents against *Biscogniauxia mediterranea* and *Diplodia**corticola*. J. Fungi.

[33] Emberger L (1932). Sur une formule climatique et ses applications en botanique. La Meteorol..

[34] Emberger, L. *Une classification biogéographique des climats*. *Recueil des Travaux des Laboratoires de Botanique* Vol 7, (1955).

[35] White TJ, Bruns T, Lee SJWT, Taylor JL (1990). Amplification and direct sequencing of fungal ribosomal RNA genes for phylogenetics. PCR Protoc. Guid. Methods Appl..

[36] Gomes T, Pereira JA, Benhadi J, Lino-Neto T, Baptista P (2018). Endophytic and epiphytic phyllosphere fungal communities are shaped by different environmental factors in a Mediterranean ecosystem. Microb. Ecol..

[37] Martins F, Mina D, Pereira JA, Baptista P (2021). Endophytic fungal community structure in olive orchards with high and low incidence of olive anthracnose. Sci. Rep..

[38] Castresana J (2000). Selection of conserved blocks from multiple alignments for their use in phylogenetic analysis. Mol. Biol. Evol..

[39] Peláez F, González V, Platas G, Sánchez-Ballesteros J, Rubio V (2008). Molecular phylogenetic studies within the Xylariaceae based on ribosomal DNA sequences. Fungal Divers..

[40] Mazzaglia A, Anselmi N, Vicario S, Vannini A (2001). Sequence analysis of the 5.8S rDNA and ITS regions in evaluating genetic relationships among some species of Hypoxylon and related genera. Mycol. Res..

[41] Miller, M. A., Pfeiffer, W. & Schwartz, T. Creating the CIPRES Science Gateway for inference of large phylogenetic trees. In *Gateway Computing Environments Workshop, GCE* 1–8 (2010) 10.1109/GCE.2010.5676129.

[42] Lanfear R, Frandsen PB, Wright AM, Senfeld T, Calcott B (2017). Partitionfinder 2: New methods for selecting partitioned models of evolution for molecular and morphological phylogenetic analyses. Mol. Biol. Evol..

[43] Ronquist F (2012). Mrbayes 3.2: Efficient Bayesian phylogenetic inference and model choice across a large model space. Syst. Biol..

[44] Trifinopoulos J, Nguyen LT, von Haeseler A, Minh BQ (2016). W-IQ-TREE: A fast online phylogenetic tool for maximum likelihood analysis. Nucleic Acids Res..

[45] Nguyen LT, Schmidt HA, Von Haeseler A, Minh BQ (2015). IQ-TREE: A fast and effective stochastic algorithm for estimating maximum-likelihood phylogenies. Mol. Biol. Evol..

[46] Kalyaanamoorthy S, Minh BQ, Wong TKF, Von Haeseler A, Jermiin LS (2017). ModelFinder: Fast model selection for accurate phylogenetic estimates. Nat. Methods.

[47] Chernomor O, Von Haeseler A, Minh BQ (2016). Terrace aware data structure for phylogenomic inference from supermatrices. Syst. Biol..

[48] Hoang DT, Chernomor O, Von Haeseler A, Minh BQ, Vinh LS (2018). UFBoot2: Improving the ultrafast bootstrap approximation. Mol. Biol. Evol..

[49] Rambaut, A., Suchard, M., Nenarokov, S. & Klötzl, F. FigTree. (2018).

[50] Stöver BC, Müller KF (2010). TreeGraph 2: Combining and visualizing evidence from different phylogenetic analyses. BMC Bioinform..

[51] Project, I. Inkscape. (2020).

[52] Peakall R, Smouse PE (2012). GenALEx 6.5: Genetic analysis in Excel. Population genetic software for teaching and research—an update. Bioinformatics.

[53] Yeh F, Boyle T (1997). Population genetic analysis of co-dominant and dominant markers and quantitative traits. Belgian J. Bot..

[54] Team, R. C. R: A language and environment for statistical computing (2019).

[55] Oksanen, J. *et al.* vegan: community ecology package (R package version 2.5-4) (2019).

[56] Chambers, J. M. SoDA: Functions and Examples for ‘Software for Data Analysis’. (2020).

[57] Carbone I, Kohn LM (1999). A method for designing primer sets for speciation studies in filamentous ascomycetes. Mycologia.

[58] Stephenson SA, Green JR, Manners JM, Maclean DJ (1997). Cloning and characterisation of glutamine synthetase from *Colletotrichum **gloeosporioides* and demonstration of elevated expression during pathogenesis on *Stylosanthes**guianensis*. Curr. Genet..

[59] Hoffman MT, Arnold AE (2008). Geographic locality and host identity shape fungal endophyte communities in cupressaceous trees. Mycol. Res..

[60] Glass NL, Donaldson GC (1995). Development of primer sets designed for use with the PCR to amplify conserved genes from filamentous ascomycetes. Appl. Environ. Microbiol..

[61] Brown AHD, Feldman MW, Nevo E (1980). Multilocus structure of natural populations of *Hordeum **spontaneum*. Genetics.

[62] Kamvar ZN, Tabima JF, Grünwald NJ (2014). *Poppr*: An R package for genetic analysis of populations with clonal, partially clonal, and/or sexual reproduction. PeerJ.

