# Supplementary material for: Phylogenetic analysis and genetic diversity of the xylariaceous ascomycete Biscogniauxia mediterranea from cork oak forests in different bioclimates
Source: Sci Rep. 2022 Feb 16;12:2646. doi: 10.1038/s41598-022-06303-7 (PMC8850622; doi:10.1038/s41598-022-06303-7)
Supplement: Supplementary file 1 — Supplementary Information. [file 41598_2022_6303_MOESM1_ESM.pdf]

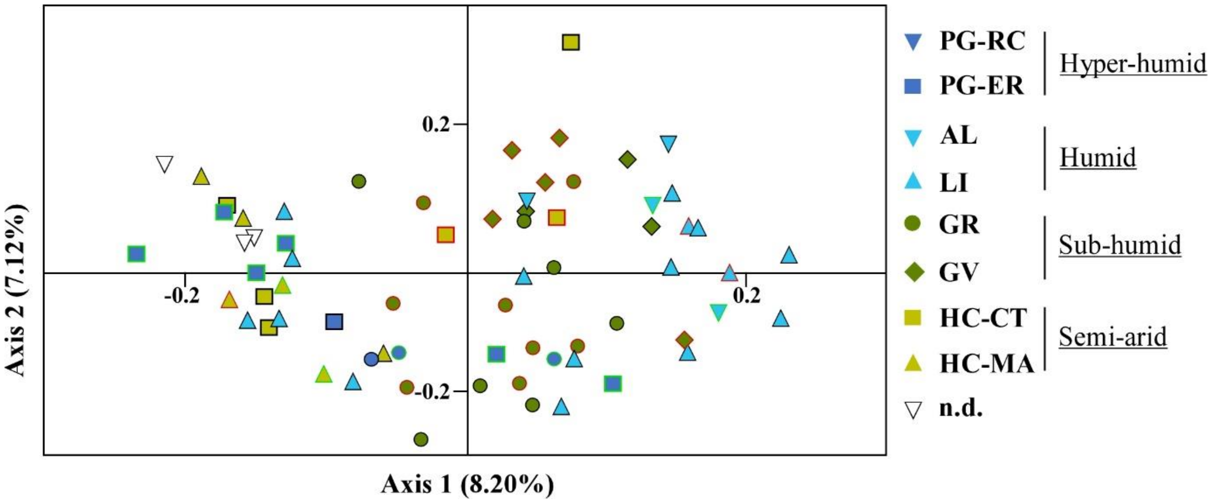

Figure S1. Principal coordinates analysis (PCoA) based on genetic distance of *B. mediterranea* obtained from different cork oak forests and bioclimates. The percentage of the total variance explained by each axis is displayed in parentheses. Symbols bordered by red line represent isolates collected from declining cork oak trees, black line by trees with mild symptoms of decline and light green line by healthy trees. Isolates from olive trees are represented by inverted triangle (n.d.).

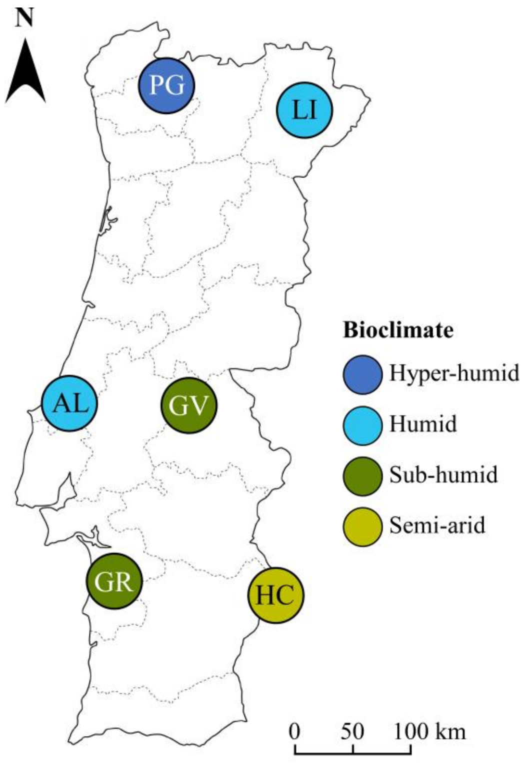

Figure S2. Distribution of sampled cork oak forests. Forests are highlighted in different colors, according to their bioclimate, as follow: dark blue for hyper-humid (PG-RC and PG-ER), light blue for humid (AL and LI), green for sub-humid (GV and GR) and dark yellow for semi-arid (HC-MA and HC-CT). Map was generated using QGIS version 3.14.16-Pi (https://qgis.org/en/site/).

Table S1. Nei's pairwise genetic distances between *B. mediterranea* populations from different bioclimates (A), forests (B), trees with different disease severity levels (C), defoliation (D), with exudates (E) and cankers (F). VAD: very accentuated defoliation; AD: accentuated defoliation; MD: moderate defoliation; LD: light defoliation; and ND: no defoliation.

A.

**Bioclimate**	Hyper-humid	Humid	Sub-humid	Semi-arid
Hyper-humid	-			
Humid	0.085	-		
Sub-humid	0.053	0.054	-	
Semi-arid	0.054	0.080	0.067	-

B.

**Forest**	**PG-ER**	**PG-RC**	**LI**	**AL**	**GV**	**GR**	**HC-CT**	**HC-MA**
PG-ER	-							
PG-RC	0.152	-						
LI	0.106	0.120	-					
AL	0.244	0.185	0.108	-				
GV	0.173	0.136	0.103	0.155	-			
GR	0.098	0.076	0.067	0.131	0.094	-		
HC-CT	0.170	0.123	0.111	0.184	0.119	0.109	-	
HC-MA	0.138	0.079	0.113	0.196	0.147	0.117	0.130	-

C.

**Disease severity level**	**Declining**	**Mild**	**Healthy**
Declining	-		
Mild	0.022	-	
Healthy	0.048	0.035	-

D.

**Defoliation**	**VAD**	**AD**	**MD**	**LD**	**ND**
VAD	-				
AD	0.200	-			
MD	0.129	0.059	-		
LD	0.123	0.070	0.019	-	
ND	0.171	0.064	0.054	0.058	-

E.

**Exudates**	**No**	**Yes**
No	-	
Yes	0.033	-

F.

**Cankers**	**No**	**Yes**
No	-	
Yes	0.048	-

Table S2. Estimation of linkage disequilibrium by index of association (I_A) and standardized index of association ($\bar{r}_d$) from cork oak Portuguese populations of *B. mediterreanea*.

Population	I_A	$\bar{r}_d$	*p-value*
**PG-ER**	-0.100	-0.003	0.405
**PG-RC**	0.321	0.006	0.161
**Hyper-humid**	-0.034	-0.001	0.532
**LI**	0.416	0.007	0.027
**AL**	0.371	0.012	0.265
**Humid**	0.463	0.007	0.004
**GV**	0.614	0.011	0.044
**GR**	0.292	0.004	0.070
**Sub-humid**	0.363	0.005	0.011
**HC-CT**	0.570	0.011	0.090
**HC-MA**	0.106	0.002	0.356
**Semi-arid**	0.530	0.008	0.022
**All**	0.233	0.003	0.001

Table S3. Data from cork oak forests, used for obtaining *B. mediterranea* isolates. Tmax corresponds to the mean maximum temperature of the hottest month, Tmin to the mean minimum temperature of the coldest month and P to mean total precipitation for the 10 years previous to sampling collection (2006-2016). Forests are highlighted in different colors as described in Figure S1.

Location	Forest	Collection date	GPS coordinates	Tmax	Tmin	P
Peneda Gerês	PG-ER	May, 2017	41° 42' N 8° 6' W	26.72	3.29	1391
	PG-RC	July, 2017	41° 45' N 8° 1' W	26.61	2.80	1378
Limãos	LI	April, 2017	41° 31' N 6° 49' W	29.44	2.29	995
Alcobaça	AL	May, 2017	39° 27' N 9° 2' W	24.70	8.04	821
Gavião	GV	July, 2017	39° 27' N 7° 55' W	30.45	6.65	844
Grândola	GR	May, 2017	38° 11' N 8° 37' W	30.34	7.31	740
Herdade Contenda	HC-CT	October, 2017	38° 2' N 7° 0.5' W	32.47	4.86	632
	HC-MA	October, 2017	38° 2' N 7° 1.9' W	32.45	4.68	641

Table S4. Disease symptoms of sampled cork trees from which *B. mediterranea* isolates were obtained. Defoliation was evaluated in a scale from 0 (no damage) to 5 (extreme damage) and other symptoms from 0 (no damage) to 3 (severe damage). Disease severity levels are presented, being determined based on disease symptoms, as described in Costa et al. (2020). Isolates obtained from healthy cork oak trees are highlighted in green, while those from declining trees are highlighted in red.

**Isolate**	**Disease symptoms**							**Disease severity level**
	**Canopy**				**Trunk**			
	**Defoliation**	**Dried**	**Wilting**	**Decolorated**	**Cankers**	**Decolorated**	**Exudates**	
**Bm02**	1	0	0	1	0	1	1	Mild
**Bm03**	1	0	0	1	0	1	1	Mild
**Bm04**	1	0	0	0	0	0	0	**Healthy**
**Bm05**	1	0	0	0	0	0	0	**Healthy**
**Bm06**	1	0	0	0	0	0	0	**Healthy**
**Bm07**	1	1	1	1	0	0	0	Mild
**Bm08**	2	1	0	2	0	1	1	**Declining**
**Bm09**	2	1	0	1	0	0	1	Mild
**Bm10**	1	0	0	0	0	1	0	Mild
**Bm11**	2	1	0	1	0	0	1	Mild
**Bm12**	1	0	0	0	0	1	0	Mild
**Bm13**	2	1	0	2	0	1	1	**Declining**
**Bm14**	1	0	0	0	0	1	0	Mild
**Bm15**	2	1	0	1	0	0	1	Mild
**Bm16**	1	0	0	0	0	1	0	Mild
**Bm17**	1	0	0	0	0	1	0	Mild
**Bm18**	1	1	1	1	0	0	0	Mild
**Bm19**	2	1	0	1	0	0	1	Mild
**Bm20**	1	0	0	0	0	1	0	Mild
**Bm21**	1	0	0	0	0	1	0	Mild
**Bm22**	2	1	0	1	0	0	0	Mild
**Bm23**	4	0	0	1	0	0	0	**Declining**
**Bm24**	1	0	0	0	0	0	0	**Healthy**
**Bm25**	1	0	0	0	0	0	0	**Healthy**
**Bm26**	2	0	0	0	0	0	0	Mild
**Bm27**	2	0	0	0	0	0	0	Mild
**Bm28**	1	0	0	0	0	0	0	**Healthy**
**Bm29**	2	0	0	1	0	0	0	Mild
**Bm30**	2	0	0	1	0	0	0	Mild
**Bm31**	1	0	0	0	0	0	0	**Healthy**
**Bm32**	0	0	0	0	0	0	0	**Healthy**
**Bm33**	1	0	0	0	0	0	0	**Healthy**
**Bm34**	0	0	0	0	1	0	0	**Healthy**
**Bm35**	1	1	0	0	1	0	0	Mild

Table S4. *continuation*

Isolate	Disease symptoms							Disease severity level
	Canopy				Trunk			
	Defoliation	Dried	Wilting	Decolorated	Cankers	Decolorated	Exudates	
**Bm36**	0	0	0	0	1	0	0	**Healthy**
**Bm37**	0	0	0	0	1	0	0	**Healthy**
**Bm38**	0	0	0	0	1	0	0	**Healthy**
**Bm39**	1	0	0	0	0	0	0	**Healthy**
**Bm41**	1	0	0	0	0	0	0	**Healthy**
**Bm42**	1	0	0	1	0	0	0	Mild
**Bm43**	2	1	1	1	0	1	1	**Declining**
**Bm44**	2	1	0	1	0	1	0	**Declining**
**Bm45**	1	0	0	1	0	1	1	Mild
**Bm46**	1	1	0	1	0	1	1	Mild
**Bm47**	1	0	0	1	0	1	1	Mild
**Bm48**	1	0	0	1	0	1	1	Mild
**Bm49**	2	1	0	1	0	1	0	**Declining**
**Bm50**	2	1	1	1	0	1	1	**Declining**
**Bm51**	1	1	0	1	0	1	1	Mild
**Bm52**	2	1	1	1	0	1	1	**Declining**
**Bm53**	4	1	1	1	0	1	1	**Declining**
**Bm54**	2	1	1	1	0	1	1	**Declining**
**Bm55**	1	0	0	1	0	1	1	Mild
**Bm56**	1	1	0	1	0	1	1	Mild
**Bm57**	2	1	0	1	0	1	0	**Declining**
**Bm58**	1	1	0	1	0	1	1	Mild
**Bm59**	2	1	1	1	0	1	1	**Declining**
**Bm60**	3	0	0	1	1	1	1	**Declining**
**Bm61**	3	0	0	1	1	1	1	**Declining**
**Bm62**	0	0	0	0	1	0	1	Mild
**Bm63**	3	0	0	1	1	1	1	**Declining**
**Bm64**	2	0	0	0	2	1	1	**Declining**
**Bm65**	0	0	0	0	1	1	1	Mild
**Bm66**	0	0	0	0	1	1	1	Mild
**Bm67**	3	0	0	1	1	1	1	**Declining**
**Bm68**	2	0	0	1	0	0	0	Mild
**Bm69**	3	0	0	0	0	0	1	**Declining**
**Bm70**	3	0	0	0	0	0	1	**Declining**
**Bm71**	2	0	0	1	0	0	0	Mild
**Bm72**	2	0	0	1	0	0	0	Mild
**Bm73**	2	0	0	1	0	0	0	Mild
**Bm74**	2	0	0	1	0	0	0	Mild
**Bm75**	3	0	0	0	0	0	1	**Declining**
**Bm76**	2	0	0	0	0	0	0	Mild
